# Stability of gene expression and epigenetic profiles highlights the utility of patient-derived paediatric acute lymphoblastic leukaemia xenografts for investigating molecular mechanisms of drug resistance

**DOI:** 10.1186/1471-2164-15-416

**Published:** 2014-06-01

**Authors:** Nicholas C Wong, Vivek A Bhadri, Jovana Maksimovic, Mandy Parkinson-Bates, Jane Ng, Jeff M Craig, Richard Saffery, Richard B Lock

**Affiliations:** Murdoch Childrens Research Institute, Royal Children’s Hospital, Flemington Road, Parkville, Victoria 3052 Australia; Ludwig Institute for Cancer Research, Olivia Newton John Cancer and Wellness Centre, Austin Hospital, Burgundy Street, Heidelberg, Victoria 3184 Australia; Children’s Cancer Institute Australia for Medical Research, Lowy Cancer Research Centre, UNSW, PO Box 81, Sydney, NSW 2052 Australia; Department of Paediatrics, The University of Melbourne, Royal Children’s Hospital, Flemington Road, Parkville, Victoria 3052 Australia

**Keywords:** Acute lymphoblastic leukaemia, Xenografts, Genome-wide DNA methylation, Microarray analysis of gene expression, Glucocorticoid resistance

## Abstract

**Background:**

Patient-derived tumour xenografts are an attractive model for preclinical testing of anti-cancer drugs. Insights into tumour biology and biomarkers predictive of responses to chemotherapeutic drugs can also be gained from investigating xenograft models. As a first step towards examining the equivalence of epigenetic profiles between xenografts and primary tumours in paediatric leukaemia, we performed genome-scale DNA methylation and gene expression profiling on a panel of 10 paediatric B-cell precursor acute lymphoblastic leukaemia (BCP-ALL) tumours that were stratified by prednisolone response.

**Results:**

We found high correlations in DNA methylation and gene expression profiles between matching primary and xenograft tumour samples with Pearson’s correlation coefficients ranging between 0.85 and 0.98. In order to demonstrate the potential utility of epigenetic analyses in BCP-ALL xenografts, we identified DNA methylation biomarkers that correlated with prednisolone responsiveness of the original tumour samples. Differential methylation of *CAPS2*, *ARHGAP21*, *ARX* and *HOXB6* were confirmed by locus specific analysis. We identified 20 genes showing an inverse relationship between DNA methylation and gene expression in association with prednisolone response. Pathway analysis of these genes implicated apoptosis, cell signalling and cell structure networks in prednisolone responsiveness.

**Conclusions:**

The findings of this study confirm the stability of epigenetic and gene expression profiles of paediatric BCP-ALL propagated in mouse xenograft models. Further, our preliminary investigation of prednisolone sensitivity highlights the utility of mouse xenograft models for preclinical development of novel drug regimens with parallel investigation of underlying gene expression and epigenetic responses associated with novel drug responses.

**Electronic supplementary material:**

The online version of this article (doi:10.1186/1471-2164-15-416) contains supplementary material, which is available to authorized users.

## Background

Despite progress in the treatment of several cancers over recent decades, the lack of clinically relevant tumour models for individual subtypes of human cancer has proven to be a major impediment in the development of effective anti-cancer therapies [1]. Approaches that facilitate development of novel rational therapies targeting specific tumours (or specific features of tumours) remain an urgent priority. Traditional models of human cancer involving the analysis of immortalised cell lines have given way in recent years to more clinically relevant studies in models that mirror the features of primary tumours [2]. The two main approaches have been the generation of primary tumour-derived cell lines, and the generation of mouse models, either via transgenic approaches or through the engraftment of primary human tumour into immune-compromised mouse models [3]. Mouse models have been used extensively in this regard, for preclinical testing of drug efficacy and toxicity prior to establishing clinical trials. A broad panel of xenografts with known treatment responsiveness, and well-defined molecular profiles, would provide an excellent adjunct to these models [4].

Mouse xenograft models of haematological malignancies, established by the transplantation of donor cells into non-obese diabetic/severe combined immunodeficient (NOD/SCID) or NOD/SCID/IL-2 receptor gamma chain^−/−^ (NSG) mice, are recognised as one of the most clinically relevant systems for investigating leukaemia biology and testing new treatments [5–12]. This is due to the faithful recapitulation of many aspects of the human disease, including kinetics of engraftment in the bone marrow (BM), with subsequent infiltration of the spleen, peripheral blood and other organs [10, 13, 14]. For these reasons, patient-derived xenografts (PDXs) are considered superior to *in vitro* immortalised cancer cell lines that show many differences to primary tumours, including gene expression, drug responsiveness and epigenetic profiles [15], which is most likely due to the selective processes associated with long term culturing. PDXs have become increasingly popular as evidence mounts that they accurately recapitulate many of the features of patient tumours, such as tumour microenvironment, differentiation state and morphology, architecture and in some instances molecular signatures of the original patient tumour (reviewed in [1, 2]).

To establish the relevance of PDX models to primary tumours, high density molecular profiling of gene expression and epigenetic markers should be performed. This was recently demonstrated for gene expression both between two tissue types, bone marrow and spleen and between independently engrafted mice for T-ALL [16].

As a first step towards examining the equivalence of epigenetic profiles between primary tumour and xenograft, we carried out parallel DNA methylation and gene expression profiling on a panel of childhood B-cell precursor acute lymphoblastic leukaemia (BCP-ALL) selected by their clinical responses to prednisolone. This panel consisted of five individuals who had a good response to prednisolone (PGR) and five who had a poor response (PPR). By comparing DNA methylation and gene expression profiles between primary and derived, single-passaged xenograft lines, we report the stability of both gene expression and DNA methylation in the xenograft, further highlighting their potential for exploring gene expression and epigenetic changes associated with responses to established and novel drugs.

## Methods

### Patient samples, characteristics and xenograft model generation

All experimental studies were approved by the Human Research Ethics Committee and the Animal Care and Ethics Committee of the University of New South Wales. Written informed consent was obtained from the parents or guardians of paediatric ALL patients for use of biopsy samples in research, with the exception of samples obtained prior to May 2003 (ALL-26, ALL-28 and ALL-53), for which a waiver had been issued by the Human Research Ethics Committee. A total of 10 xenograft lines were generated from children diagnosed with BCP-ALL. Individuals were selected based on their response to prednisolone. We classified prednisolone poor responders (PPR) as patients with a peripheral blast count of ≥ 1 × 10^9^/L on day 8 following induction treatment with prednisolone and a single intrathecal dose of methotrexate, while a prednisolone good responder (PGR) demonstrated a day 8 peripheral blast count of < 1 × 10^9^/L (Table 1). Xenografts were established in NOD/SCID or NSG mice using direct explants of patient BM biopsies, exactly as described previously [10, 17]. When mice were highly engrafted with leukaemia human CD45^+^, mononuclear cells were isolated from spleens by FACS at >90% purity and cryopreserved for subsequent experiments.

**Table 1:** **Patient demographics of xenografts used in this study**

	Xenograft	Sex	Age	Cytogenetics	Immunophenotype	Diagnosis WCC	Diagnosis blasts	Day 8 blasts
			(Months)		(Diagnostic patient sample)	×10^9^/L	×10^9^/L	×10^9^/L
**PPR**	ALL-28	M	20	Hyperdiploid	CD45-/DR+/10+/19+/2-/7-/13-/33+/34+	15.0	11.8	**1.9**
**PPR**	ALL-50	M	131	Normal	CD45+/DR+/10+/19+/20+	34.6	26.1	**5.5**
**PPR**	ALL-54	M	89	Normal	CD45+/DR+/10+/19+/20+/34+/13-/33-	185.0	174.8	**1.2**
**PPR**	ALL-55	M	176	t(9;22)	CD45+/DR+/10+/19+/13+/33+/34+	422.5	388.7	**22.6**
**PPR**	ALL-57	F	72	t(1;19)	CD45+/DR+/19+/10+/34-2-/7-	15.9	7.2	**1.6**
**PGR**	ALL-26	F	43	t(12;21)	CD45+/DR+/CD19+/10+/22+/3-/34+/117-/Cu-/TdT+	89.4	80.5	**0.0**
**PGR**	ALL-51	M	19	dic(7;9)	CD45+/DR+/CD19+/10+/22+/34-/117-/Cu-/TdT+	90.5	76.9	**0.0**
**PGR**	ALL-52	M	138	t(7;15)	CD45+/DR+/CD19+/22+/13+/33+/10-/34-/Cu-/TdT+	14.4	4.0	**0.0**
**PGR**	ALL-53	M	87	t(12;21)	CD45+/DR+/10+/19+/34+	20.3	13.8	**0.1**
**PGR**	ALL-56	M	120	t(9;22)	CD45-/DR+/10+/19+/34+/2-/7-/13-/33-	8.5	0.1	**0.0**

### Genomic DNA and total RNA extraction

Genomic DNA was extracted from the primary bone marrow biopsies used for xenografting and from cells harvested from the spleens of engrafted animals for each xenograft using standard phenol/chloroform extraction and isopropanol precipitation. Total RNA was extracted using TriZol Reagent (Life Technologies, Carlsbad, USA) according to manufacturer’s instructions. Quality and yield were measured using a Nanodrop spectrophotometer.

### Sodium bisulphite conversion of genomic DNA

Genomic DNA was converted for DNA methylation analysis using the MethylEasy Xceed Kit (Human Genetic Signatures, Sydney, Australia) according to manufacturer’s instructions. Converted DNA was used for downstream Illumina Infinium DNA methylation BeadArray analysis and SEQUENOM EpitTYPER validation.

### Genome-scale DNA methylation analysis

Converted genomic DNA was processed and analysed for Illumina Infinium HumanMethylation27 BeadArray (Illumina, San Diego, USA) according to manufacturer’s instructions (ServiceXS, Leiden, The Netherlands). This BeadArray platform interrogates 27,578 CpG sites across the human genome. The arrays were scanned using an Illumina BeadArray Reader and subsequently processed using the Illumina GenomeStudio V.1 software package. The Bioconductor Lumi package was used for downstream data processing and normalisation [18]. Briefly, DNA probe methylation data were quality checked and then colour balance adjusted, background corrected and scaled based on the mean of all probes, using the methylation simple scaling normalization (SSN) implemented within the Lumi package. CpG sites with at least one sample having a detection p-value > 0.01 were excluded from subsequent analyses, leaving 27,341 CpG sites. Differential methylation analysis was performed using the LIMMA package from Bioconductor [19]. Significantly differentially methylated probes were selected based on a Benjamini-Hochberg adjusted p-value < 0.05. The methylation microarray data have been deposited into Gene Expression Ominibus (http://www.ncbi.nlm.nih.gov/geo/) with the identifier GSE57581.

### Gene expression Illumina array analysis

Total RNA was extracted from the primary and xenograft tumours and amplified using the Illumina TotalPrep RNA amplification kit (Ambion, Austin, USA). The amplified total RNA was analysed using Illumina WG-6_V3 chips (Illumina, San Diego, USA) according to manufacturer’s instructions. The sample probe profiles with no normalisation or background correction were exported from BeadStudio (version 3.0.14, Illumina), and the data were pre-processed using quantile normalisation. Probes with detection p-value greater than 0.01 on all arrays were deemed as non-expressed probes and filtered out. Differential gene expression was determined using LIMMA with the positive False Discovery Rate (FDR) correction for multiple testing (Benjamini-Hochberg adjusted p-value < 0.05). The gene expression microarray data have been deposited into Gene Expression Ominibus (http://www.ncbi.nlm.nih.gov/geo/) with the identifier GSE57491.

### SEQUENOM MassArray EpiTYPER analysis

Primers (detailed in Additional file 1: Table S1) were designed to generate PCR amplicons from bisulphite converted genomic DNA suitable for SEQUENOM EpiTYPER chemistry as per the manufacturer’s protocol. Samples were analysed using MALDI-TOF mass spectrometry, DNA methylation information was collected using EpiTYPER Viewer Software (v 1.0.5). Non-analysable and poor quality CpG sites were removed from downstream analysis as previously described [20].

## Results

### Xenograft models of BCP-ALL are an accurate reflection of DNA methylation and gene expression status of the corresponding primary tumour

One sample in our analysis, ALL28P, failed to meet array quality metrics (low overall signal intensity). Therefore, the matching xenograft pair, ALL28X along with ALL28P gene expression data was removed from subsequent analysis. ALL28 was also removed from the DNA methylation and gene expression correlation analysis herein.

**Figure 1:**
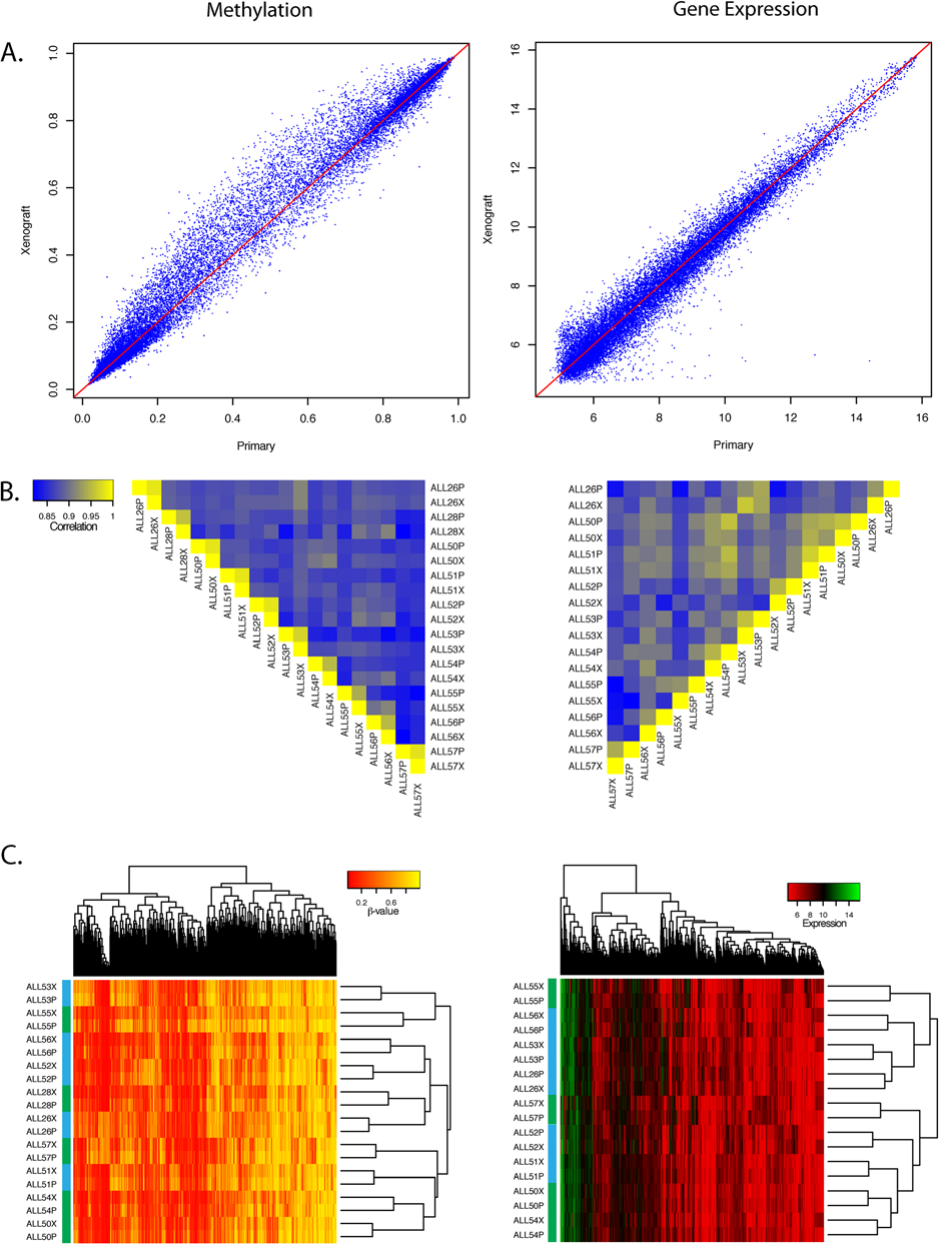
**Comparisons of DNA methylation and gene expression profiles between primary tumour tissue and xenografts. (A)** Scatterplots of DNA methylation and gene expression array results from ALL26 showing high correlation between primary and xenograft tumours. **(B)** Heatmap plot of Pearson’s correlation coefficients of all primary and xenograft samples analysed for DNA methylation and gene expression. Coefficients greater than 0.94 and 0.84 between matching primary and xenograft tumours were observed for DNA methylation and gene expression respectively. **(C)** Heatmap plot of the most variable DNA methylation and gene expression probes. A high level of similarity between matching primary and xenograft tumours resulted in all pairs clustering together. Green Sample Bar depicts PPR, Blue sample Bar depicts PGR.

Plotting the beta values of the entire data set revealed similar DNA methylation profiles between primary tumour tissue and the matching xenograft from each of the 10 patients in our study. Similarly, gene expression levels between primary tumour tissue and xenograft were also comparable (Figure 1A). For genome-scale DNA methylation, Pearson’s correlation coefficients between matching primary and xenograft samples ranged between 0.94-0.98 while correlation coefficients between individuals ranged between 0.80-0.91. For genome-wide gene expression, Pearson’s correlation coefficients between primary and xenograft samples ranged between 0.85-0.97 and between individuals was greater than 0.83-0.96 (Figure 1B). Gene expression profiles between individuals were more correlated than their DNA methylation profiles.

Consistent with this observation, unsupervised hierarchical clustering of the most variable DNA methylation and gene expression across all samples revealed clustering of matching primary and xenograft samples. This implies that the profiles from the xenografts recapitulate the profile of the primary tumour (Figure 1C).

To identify differential DNA methylation between primary tumour and matching xenograft samples we applied a linear model with empirical Bayes estimation and found 1564 probes to be differentially methylated between matching primary tumour and xenograft sample after correction for multiple testing (adjusted p-value < 0.05, Additional file 2: Table S2). The majority of these probes demonstrated a small change in DNA methylation with the average difference across individuals ranging from 0.4 to 8.6% (Additional file 3: Figure S1A).

We also looked for differential gene expression between matching primary and xenograft cell lines again applying a linear model with empirical Bayes estimation on the genome-scale gene expression microarray results. We found 3441 probes from 3208 genes to be differentially expressed between primary and xenograft lines (adjusted p-value < 0.05, Additional file 4: Table S3). However, as we observed with DNA methylation, the differences in expression of these probes between primary and xenograft were minimal with an average fold difference in expression between primary and xenograft tumours of 1.12 (Additional file 3: Figure S1B).

Using DAVID (http://david.abcc.ncifcrf.gov/), the differentially methylated and differentially expressed genes between primary and matching xenograft lines were found to be mainly involved in haematological and cell signalling processes that could be accounted for given the cellular origins of the primary (bone marrow) and xenograft (spleen) samples.

Given the relatively small number of differentially methylated probes (6%) and differentially expressed probes (17%), and the minimal absolute differences in DNA methylation and expression (Additional file 3: Figure S1A and S1B), our results indicate that xenograft models largely recapitulate the DNA methylation and gene expression profile of the corresponding primary tumour. This highlights the potential utility of xenograft cell lines for modelling primary disease.

### Molecular biomarkers associated with prednisolone response

**Figure 2:**
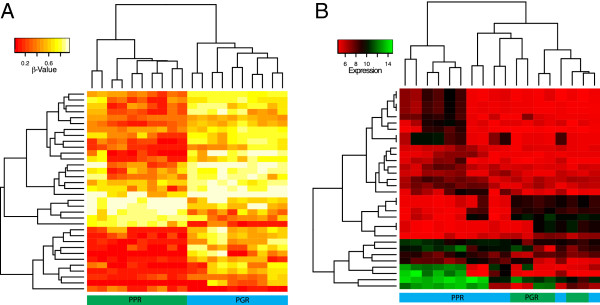
**Heatmap plot of the most significant DNA methylation and gene expression probes distinguishing prednisolone good responders (PGR) from poor responders (PPR) after LIMMA analysis (BH adjusted p-value < 0.05).** DNA methylation probes distinguished PGR from PPR while gene expression probes did not.

We then sought to identify differential DNA methylation and gene expression associated with prednisolone poor (PPR) and prednisolone good (PGR) responders and included primary and xenograft samples in our analysis. After correction for multiple testing, 35 DNA methylation probes were differentially methylated between PPR and PGR (Benjamini-Hochberg adjusted p-value < 0.05, Table 2, Figure 2). Gene expression analysis revealed 23 genes differentially expressed between PPR and PGR (Benjamini-Hochberg adjusted p-value < 0.05, Table 2, Figure 2). From these lists, we did not find any commonly annotated genes associated with prednisolone response between the top differentially methylated and top differentially expressed probes. Differential DNA methylation segregated PPR from PGR by supervised hierarchical clustering and may serve as potential biomarkers for prednisolone response (Figure 2A). However, interrogating gene expression alone did not accurately segregate PPR from PGR (Figure 2B). Functional annotation of differentially methylated genes annotated to these probes identified a number of apoptotic, cell signalling/structure pathways that did not reach statistical significance (Additional file 5: Table S4).

**Table 2:** **Differential probes associated with prednisolone response**

Probe type	Probe ID	Gene symbol	Adjusted p-value	ACC	DESC
DNA methylation probes	cg02780988	KRTHA6	0.0001	NM_003771	keratin 36
	cg16848873	HOXB6	0.0001	NM_018952	homeobox B6
	cg00546897	LOC284837	0.0002	NM_194310	
	cg02789485	MGC39497	0.0007	NM_152436	GLI pathogenesis-related 1 like 2
	cg01605783	LOC284837	0.0010	NM_194310	
	cg20291222	CAPS2	0.0012	NM_032606	calcyphosine 2
	cg05724065	PHKG1	0.0019	NM_006213	phosphorylase kinase, gamma 1 (muscle)
	cg00645579	IRF7	0.0033	NM_001572	interferon regulatory factor 7
	cg02100629	AMID	0.0158	NM_032797	apoptosis-inducing factor, mitochondrion-associated, 2
	cg20649991	LILRB5	0.0170	NM_006840	leukocyte immunoglobulin-like receptor, subfamily B (with TM and ITIM domains), member 5
	cg11952714	SNX7	0.0170	NM_015976	sorting nexin 7
	cg20050826	K6IRS2	0.0170	NM_080747	keratin 72
	cg21306775	FLJ44881	0.0190	NM_207461	
	cg20468883	BNIP2	0.0202	NM_004330	BCL2/adenovirus E1B 19kDa interacting protein 2
	cg08739282	DHX15	0.0202	NM_001358	DEAH (Asp-Glu-Ala-His) box polypeptide 15
	cg03172991	NFIX	0.0211	NM_002501	nuclear factor I/X (CCAAT-binding transcription factor)
	cg19238840	GP2	0.0211	NM_001007240	glycoprotein 2 (zymogen granule membrane)
	cg10148841	ROBO4	0.0223	NM_019055	roundabout homolog 4, magic roundabout (Drosophila)
	cg09892390	ARHGAP21	0.0254	NM_020824	Rho GTPase activating protein 21
	cg05961212	ADPRH	0.0254	NM_001125	ADP-ribosylarginine hydrolase
	cg22844623	GJA12	0.0254	NM_020435	gap junction protein, gamma 2, 47kDa
	cg18096388	PDCD1	0.0255	NM_005018	programmed cell death 1
	cg05921324	APOA4	0.0255	NM_000482	apolipoprotein A-IV
	cg13633560	LRRC32	0.0270	NM_005512	leucine rich repeat containing 32
	cg19573166	SLC22A17	0.0270	NM_020372	solute carrier family 22, member 17
	cg01410472	CRISPLD1	0.0277	NM_031461	cysteine-rich secretory protein LCCL domain containing 1
	cg26624914	AQP3	0.0377	NM_004925	aquaporin 3 (Gill blood group)
	cg23752985	VAMP8	0.0389	NM_003761	vesicle-associated membrane protein 8 (endobrevin)
	cg21148892	CLEC4F	0.0389	NM_173535	C-type lectin domain family 4, member F
	cg00032666	CXorf6	0.0402	NM_005491	mastermind-like domain containing 1
	cg19511844	ORMDL3	0.0418	NM_139280	ORM1-like 3 (S. cerevisiae)
	cg16127900	GPRC6A	0.0466	NM_148963	G protein-coupled receptor, family C, group 6, member A
	cg12552392	NFS1	0.0475	NM_181679	
	cg22437699	ARX	0.0479	NM_139058	aristaless related homeobox
	cg02849695	CCDC19	0.0486	NM_012337	coiled-coil domain containing 19
Gene expression probes	ILMN_1806907	PAWR	0.0000	NM_002583	PRKC, apoptosis, WT1, regulator
	ILMN_1794046	MTX2	0.0119	NM_006554 NM_001006635	Metaxin 2
	ILMN_1758128	CYGB	0.0119	NM_134268	Cytoglobin
	ILMN_1738438	MAST4	0.0157	NM_198828	Microtubule associated serine/threonine kinase family member 4
	ILMN_1789384	QSOX2	0.0190	NM_181701	Quiescin Q6 sulfhydryl oxidase 2
	ILMN_2306565	MTX2	0.0190	NM_006554 NM_001006635	Metaxin 2
	ILMN_2295987	NBPF1	0.0237	NM_017940	Neuroblastoma breakpoint family, member 1
	ILMN_1765772	MYO3A	0.0305	NM_017433	Myosin IIIA
	ILMN_1713934	LITAF	0.0305	NM_004862	Lipopolysaccharide-induced TNF factor
	ILMN_1668125	MYRIP	0.0305	NM_015460	Myosin VIIA and Rab interacting protein
	ILMN_1681888	PRKAR2A	0.0305	NM_004157	Protein kinase, cAMP-dependent, regulatory, type II, alpha
	ILMN_2184966	ZHX2	0.0305	NM_014943	Zinc fingers and homeoboxes 2
	ILMN_1706505	COL5A1	0.0305	NM_000093	Collagen, type V, alpha 1
	ILMN_1656057	PLAU	0.0305	NM_002658	Plasminogen activator, urokinase
	ILMN_1761540	SEMA3F	0.0305	NM_004186	Sema domain, immunoglobulin domain (Ig), short basic domain, secreted, (semaphorin) 3F
	ILMN_1753143	DKFZp761L1918	0.0305	NM_033103	Homo sapiens rhophilin-like protein mRNA, complete cds.
	ILMN_2148944	ADCY4	0.0305	NM_139247	Adenylate cyclase 4
	ILMN_1812618	ARAP3	0.0305	NM_022481	ArfGAP with RhoGAP domain, ankyrin repeat and PH domain 3
	ILMN_1681081	AGPAT2	0.0359	NM_006412	1-acylglycerol-3-phosphate O-acyltransferase 2 (lysophosphatidic acid acyltransferase, beta)
	ILMN_1743275	SH3RF3	0.0359	NM_001099289	SH3 domain containing ring finger 3
	ILMN_1656951	APCDD1	0.0359	NM_153000	Adenomatosis polyposis coli down-regulated 1
	ILMN_1719756	ZAP70	0.0359	NM_207519 NM_001079	Zeta-chain (TCR) associated protein kinase 70kDa
	ILMN_1768732	SPAG16	0.0437	NM_024532 NM_001025436	Sperm associated antigen 16

**Figure 3:**
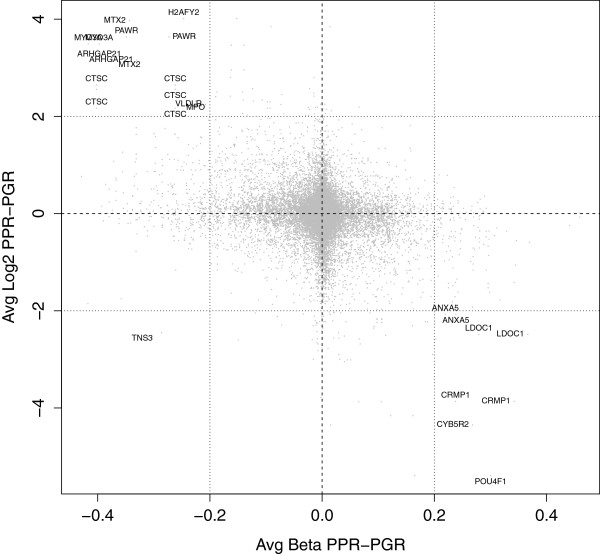
**Scatterplot of the average DNA methylation and gene expression difference between PPR and PGR samples reveals 20 genes with a negative association between gene expression and DNA methylation.**

We then determined the relationship between DNA methylation and gene expression in association with prednisolone response. Plotting the average DNA methylation and gene expression differences between PPR and PGR revealed 22 probes annotated to 12 genes that were more highly expressed and less methylated in PPR samples compared to PGR samples (gene expression cut-off greater than 2 and a DNA methylation cut-off of less than −0.2, Figure 3, Table 3). Conversely, 11 probes annotated to 8 genes were less highly expressed and more methylated in PPR samples compared to PPR (gene expression cut-off of less than −2 and a DNA methylation cut-off greater than 0.2, Figure 3, Table 3). With the exception of expression probes annotated to *PAWR*, *MTX2* and *MYO3A* no other gene expression and DNA methylation probes reached statistical significance (Table 3). DNA methylation probes associated with *PAWR*, *MTX2* and *MYO3A* demonstrated an average difference of >0.2 between groups but did not reach significance with LIMMA analysis between PPR and PGR.

**Table 3:** **Probes both differentially methylated and expressed in association with prednisolone response**

			Expression	Methylation		
Threshold	Gene_symbol.x	Gene_description.x	adj.P.Val	adj.P.Val	methDiff	expDiff
Upregulated and less methylated in PPR	H2AFY2	H2A histone family, member Y2	0.1173	0.1782	-0.2204	4.0116
(>2-fold expression, <-0.2 methylation)	H2AFY2	H2A histone family, member Y2	0.1173	0.3973	-0.3007	4.0116
	MTX2	Metaxin 2	0.0190	0.3673	-0.4061	3.9695
	PAWR	PRKC, apoptosis, WT1, regulator	0.0000	0.3154	-0.4185	3.6350
	PAWR	PRKC, apoptosis, WT1, regulator	0.0000	0.3973	-0.3026	3.6350
	MYO3A	Myosin IIIA	0.0305	0.2944	-0.4156	3.4893
	MYO3A	Myosin IIIA	0.0305	0.3154	-0.4006	3.4893
	MTX2	Metaxin 2	0.0119	0.3673	-0.4061	3.1908
	BX537570		0.2492	0.0333	-0.3571	3.1574
	BX537570		0.2492	0.1707	-0.4761	3.1574
	CTSC	Cathepsin C	0.0624	0.1782	-0.4239	2.6472
	CTSC	Cathepsin C	0.0624	0.2267	-0.2834	2.6472
	CTSC	Cathepsin C	0.0766	0.1782	-0.2834	2.5574
	CTSC	Cathepsin C	0.0766	0.2267	-0.4239	2.5574
	MOSC1	MOCO sulphurase C-terminal domain containing 1	0.0578	0.5189	-0.2051	2.5035
	NGFRAP1	Nerve growth factor receptor (TNFRSF16) associated protein 1	0.4360	0.4775	-0.2504	2.4066
	MARCKS	Myristoylated alanine-rich protein kinase C substrate	0.5467	0.4154	-0.2603	2.3418
	MPO	Myeloperoxidase	0.5920	0.4097	-0.2226	2.3130
	CTSC	Cathepsin C	0.0578	0.1782	-0.2834	2.1699
	CTSC	Cathepsin C	0.0578	0.2267	-0.4239	2.1699
	CCR7	Chemokine (C-C motif) receptor 7	0.2918	0.4434	-0.2264	2.0570
	PLS3	Plastin 3 (T isoform)	0.6662	0.3610	-0.2919	2.0256
Downregulated and more methylated in PPR	POU4F1	POU class 4 homeobox 1	0.1004	0.4404	0.3017	-5.3928
(<-2-fold expression, >0.2 methylation)	CYB5R2	Cytochrome b5 reductase 2	0.1734	0.5066	0.2699	-4.3486
	TMED6	Transmembrane emp24 protein transport domain containing 6	0.0504	0.5271	0.2317	-4.1584
	CRMP1	Collapsin response mediator protein 1	0.3007	0.4431	0.3668	-3.8640
	CRMP1	Collapsin response mediator protein 1	0.3007	0.3996	0.2607	-3.8640
	IRX3	Iroquois homeobox 3	0.5401	0.4957	0.2710	-2.9003
	LDOC1	Leucine zipper, down-regulated in cancer 1	0.2608	0.6055	0.2578	-2.4859
	DSC3	Desmocollin 3	0.5401	0.4585	0.3377	-2.2248
	DSC3	Desmocollin 3	0.5401	0.4402	0.3561	-2.2248
	ANXA5	Annexin A5	0.5923	0.3699	0.2228	-2.0708
	ANXA5	Annexin A5	0.5923	0.3727	0.2386	-2.0708

### Validation of DNA methylation biomarkers associated with prednisolone response

**Figure 4:**
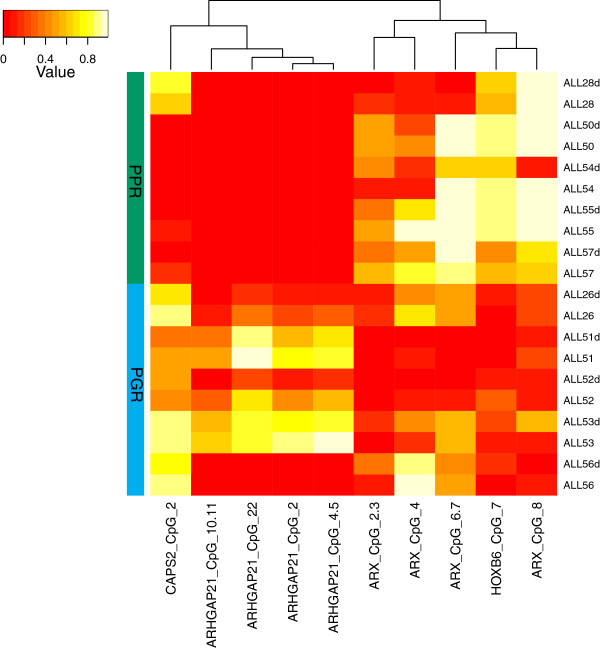
**Validation of DNA methylation across four probes,** ***CAPS2, ARHGAP21, ARX*** **and** ***HOXB6*** **using SEQUENOM EpiTYPER chemistry.**

From our array analysis, the DNA methylation changes segregated samples by prednisolone response. We validated 17 of these probes using SEQUENOM EpiTYPER chemistry on both primary and xenograft samples by selecting from our LIMMA analysis, those also associated with changes in gene expression (Additional file 6: Figure S2). Of the assays containing the 17 probes of interest, 4 regions continued to discriminate samples according to prednisolone response (Figure 4). These were associated with the genes *CAPS2 and ARHGAP21* (less methylated in PPR)*, ARX* and *HOXB6* (more methylated in PPR). Primary and matching xenograft samples showed similar DNA methylation levels in all cases.

## Discussion

It is becoming clear that the complexity of genetic, epigenetic, and subsequent gene expression disruption associated with human cancer is immense. As such, many mouse models of tumourigenesis are limited in their capacity to faithfully mimic human disease. In light of this, patient derived tumour tissue xenograft models are increasingly recognised as offering the most robust approach for testing tumour responses to various chemotherapeutic regimens, evaluating the efficacy of novel therapeutic agents, analysing the process of tumour progression at the cellular and molecular level and the identification of new therapeutic targets [2]. However, as with most mouse xenograft models, the stability of molecular profiles (gene expression and epigenetic) that regulate all aspects of tumour function remains to be determined. Confirmation of this stability is crucial in order identify molecular responses to treatment within the xenograft that could be extrapolated back to patients.

Here, we have determined the stability of genome wide DNA methylation and gene expression profiles between primary tumour cells and matching xenograft tumour cells from a small number of paediatric ALL cases with differential response to prednisolone. A high correlation in both DNA methylation and gene expression profiles was observed in all cases, confirming the stability of these molecular features of primary tumours in the mouse system. Differences in DNA methylation and gene expression between primary and xenograft samples were negligible in magnitude (Additional file 3: Figure S1) and comprised of a small fraction of probes for each array platform. The differentially methylated genes include *MYOD1, GPR6 and SLC27A6* (Table 1). Many genes associated with minor expression differences were part of the globin gene family and genes involved in oxygen transport and include *HBB, AHSP, HBD, HBA2* (Table 2). This is likely to have arisen by the differences in cellular composition as the primary tumour samples contained a milieu of haematopoietic cells, including human erythrocytes that were absent in the xenograft samples that comprised of mononuclear cells derived from the murine spleen. Given the high degree of correlation and clustering of matching primary and xenograft samples after unsupervised hierarchical clustering of the most varied probes for DNA methylation and gene expression, the xenografts described in this study are an accurate reflection of their corresponding primary tumours.

While a number of candidate genes whose DNA methylation and/or gene expression status were associated with prednisolone response, given the small sample numbers and inherent genetic heterogeneity of the tumours, the significance of these genes remains unclear. Using hierarchical clustering, the most significant probes for DNA methylation discriminated prednisolone response while the gene expression probes did not (Figure 2), reflecting the more variable nature of gene expression compared to DNA methylation [21, 22]. Using SEQUENOM, we were able to replicate DNA methylation changes at four genes associated with prednisolone response indicative of a potential DNA methylation biomarker. Taking methylation and expression status together, 20 genes were differentially regulated between good and poor responders to prednisolone (Table 3). While the genes were found to be part of apoptotic and cell signalling pathways, their significance remains unclear given the small numbers in each group. *PAWR* demonstrated significant overexpression and hypomethylation across PPRs compared to PGRs. This is a *WT1* interacting protein that also functions as a transcriptional repressor with pro-apoptotic functions and tumour resistance [23]. While the down regulation of *PAWR* confers poor prognosis in a range of solid tumours [24, 25], its role in haematological malignancy is less clear, with expression detectable in a range of leukaemias [26]. Our results warrant further investigation of *PAWR* to determine a potential role in prednisolone response and responses to other novel drug regimens in an expanded xenograft cohort.

Another gene with potential interest is *POU4F1*, which appears to be differentially regulated according to prednisolone response (Table 3). However in our analysis, statistical significance was not achieved with the modest sample size of our panel. *POU4F1* has been shown to have a role in regulating the expression of B-cell markers in t(8;21) positive acute myeloid leukaemia [27–29]. Its role in B-cell ALL response to prednisolone remains unclear and could be a potential gene target for further characterisation in an expanded B-cell ALL xenograft panel.

While our study did not identify statistically significant genes associated with prednisolone response, we present here a first pass analysis using low-resolution microarray platforms to interrogate DNA methylation and gene expression across our model system. We demonstrate that our B-cell ALL xenograft panel recapitulates the DNA methylation and gene expression profiles of the primary tumour and will facilitate future genome-wide interrogation of gene expression and DNA methylation using next generation sequencing methodology.

## Conclusions

Patient-derived tumour xenograft models offer superior utility as preclinical models over cell line systems with their ability to recapitulate the milieu and microenvironment of the primary tumour. However, the extent of gene expression and epigenetic stability within the xenograft has remained unclear at least in the haematological setting. We have demonstrated that the gene expression and DNA methylation profiles of cells taken from the spleens of engrafted mice are highly correlated to the original primary tumour. Given the similarity to the primary tumour, our study confirms the opportunity to investigate gene expression and DNA methylation biomarkers in response to novel treatment strategies.

### Availability of supporting data

The data sets supporting the results of this article are included within the article and its additional files. All microarray data presented in this paper have been deposited into Gene Expression Omnibus (http://www.ncbi.nlm.nih.gov/geo/) with the identifiers GSE57581 and GSE57491.

## Electronic supplementary material

Additional file 1: Table S1: SEQUENOM EpiTYPER primers used in this study. (XLSX 32 KB)

Additional file 2: Table S2: Differentially methylated probes between primary and xenograft tumours. (XLS 534 KB)

Additional file 3: Figure S1: Heatmap plot of the most significant DNA methylation (A) and gene expression (B) probes differentiating primary to xenograft tumours. While the samples clustered accordingly, the magnitude of DNA methylation and gene expression differences across these probes were minimal. (PDF 2 MB)

Additional file 4: Table S3: Differentially expressed probes between primary and xenograft tumours. (XLSX 1015 KB)

Additional file 5: Table S4: DAVID ontology list of functional pathways of genes found to be associated with prednisolone response. (XLSX 57 KB)

Additional file 6: Figure S2: SEQUENOM Validation of 17 probes identified as significantly differentially methylated between primary and xenograft tumours. The green side column depicts PGR samples, while red depicts PPR samples. DNA methylation of these probes were able to separate tumours on prednisolone response, with 4 (depicted in Figure 4) giving the most discriminatory power. (PDF 174 KB)
