# Supplementary figures and images for: Stability of gene expression and epigenetic profiles highlights the utility of patient-derived paediatric acute lymphoblastic leukaemia xenografts for investigating molecular mechanisms of drug resistance
Source: BMC Genomics. 2014 Jun 1;15(1):416. doi: 10.1186/1471-2164-15-416 (PMC4057609; doi:10.1186/1471-2164-15-416)

A.

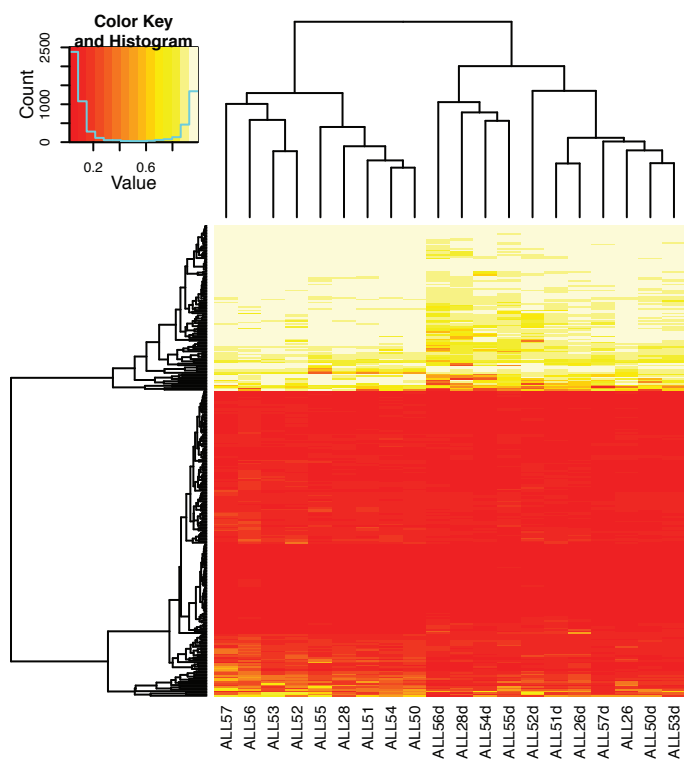

B.

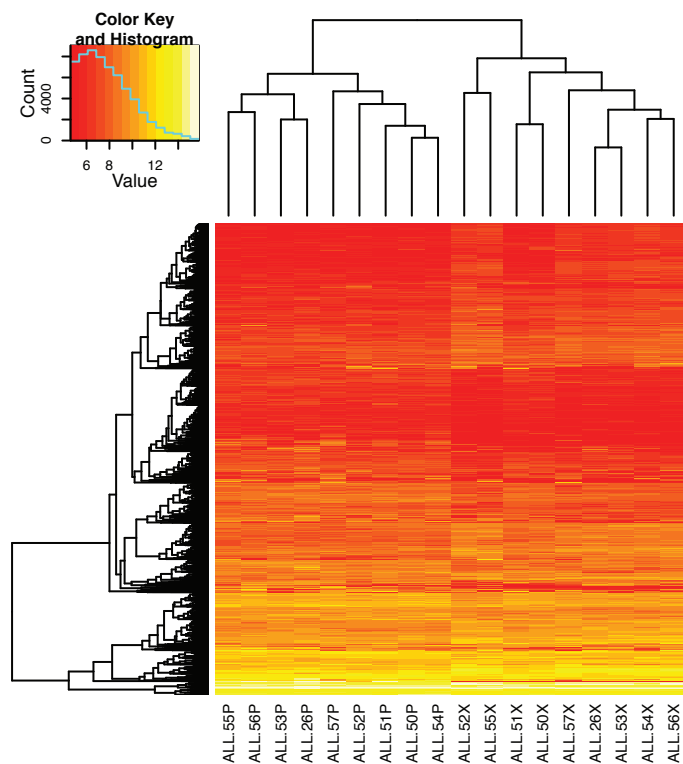

Supplement: Supplementary file 3 — Additional file 3: Figure S1: Heatmap plot of the most significant DNA methylation (A) and gene expression (B) probes differentiating primary to xenograft tumours. While the samples clustered accordingly, the magnitude of DNA methylation and gene expression differences across these probes were minimal. (PDF 2 MB) [file 12864_2013_6106_MOESM3_ESM.pdf]

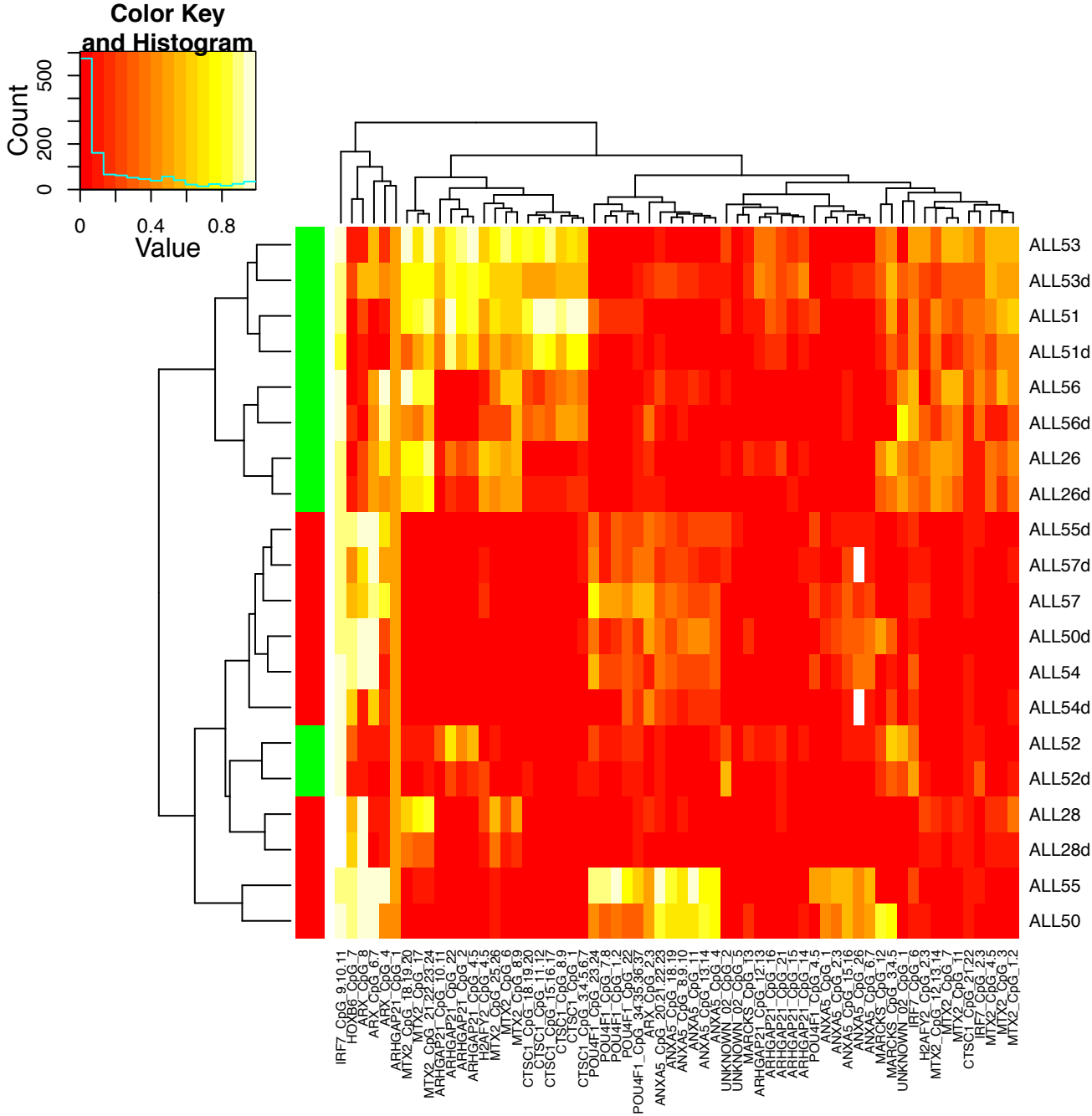

Supplement: Supplementary file 6 — Additional file 6: Figure S2: SEQUENOM Validation of 17 probes identified as significantly differentially methylated between primary and xenograft tumours. The green side column depicts PGR samples, while red depicts PPR samples. DNA methylation of these probes were able to separate tumours on prednisolone response, with 4 (depicted in Figure 4) giving the most discriminatory power. (PDF 174 KB) [file 12864_2013_6106_MOESM6_ESM.pdf]
